# Efficient strategies based on behavioral and electrophysiological methods for epilepsy-related gene screening in the *Drosophila* model

**DOI:** 10.3389/fnmol.2023.1121877

**Published:** 2023-04-20

**Authors:** Chu-Qiao Liu, Xiao-Chong Qu, Ming-Feng He, De-Hai Liang, Shi-Ming Xie, Xi-Xing Zhang, Yong-Miao Lin, Wen-Jun Zhang, Ka-Chun Wu, Jing-Da Qiao

**Affiliations:** ^1^Department of Neurology, Institute of Neuroscience, Key Laboratory of Neurogenetics and Channelopathies of Guangdong Province and the Ministry of Education of China, The Second Affiliated Hospital, Guangzhou Medical University, Guangzhou, China; ^2^The Second Clinical Medicine School of Guangzhou Medical University, Guangzhou, China; ^3^The First Clinical Medicine School of Guangzhou Medical University, Guangzhou, China; ^4^School of Clinical Medicine, LKS Faculty of Medicine, The University of Hong Kong, Hong Kong, Hong Kong SAR, China

**Keywords:** epilepsy, trio-based whole-exome sequencing, *Drosophila*, Gal4/UAS system, electrophysiology

## Abstract

**Introduction:**

With the advent of trio-based whole-exome sequencing, the identification of epilepsy candidate genes has become easier, resulting in a large number of potential genes that need to be validated in a whole-organism context. However, conducting animal experiments systematically and efficiently remains a challenge due to their laborious and time-consuming nature. This study aims to develop optimized strategies for validating epilepsy candidate genes using the *Drosophila* model.

**Methods:**

This study incorporate behavior, morphology, and electrophysiology for genetic manipulation and phenotypic examination. We utilized the Gal4/UAS system in combination with RNAi techniques to generate loss-of-function models. We performed a range of behavioral tests, including two previously unreported seizure phenotypes, to evaluate the seizure behavior of mutant and wild-type flies. We used Gal4/UAS-mGFP flies to observe the morphological alterations in the brain under a confocal microscope. We also implemented patch-clamp recordings, including a novel electrophysiological method for studying synapse function and improved methods for recording action potential currents and spontaneous EPSCs on targeted neurons.

**Results:**

We applied different techniques or methods mentioned above to investigate four epilepsy-associated genes, namely *Tango14*, *Klp3A*, *Cac*, and *Sbf*, based on their genotype-phenotype correlation. Our findings showcase the feasibility and efficiency of our screening system for confirming epilepsy candidate genes in the Drosophila model.

**Discussion:**

This efficient screening system holds the potential to significantly accelerate and optimize the process of identifying epilepsy candidate genes, particularly in conjunction with trio-based whole-exome sequencing.

## 1. Introduction

Epilepsy is a prevalent neurological disorder affecting approximately 50 million people worldwide, as reported by the World Health Organization [WHO] (2017). Although up to 30% of epilepsy cases are claimed to have a genetic cause (Weber et al., 2017), the underlying genetic factors remain unknown for a significant number of patients (Weber et al., 2017; Lasko and Luthy, 2021). The trio (two parents and the affected proband)-based study combined with whole-exome sequencing (WES) has emerged as a popular method to identify new epilepsy candidate genes (Dupépé et al., 2019). WES produces a thorough yet precise list of candidate genes at a reasonable price by focusing on the protein-coding regions (Harnish et al., 2019; Qiao et al., 2022a). Although several large-scale genetic diagnostic tests using trio-based WES have yielded significant results (Guan et al., 2021; Zhai et al., 2021), the approach has a high rate of false positive results, like another next-generation sequencing (NGS) techniques (Zhang et al., 2015), highlighting the need for direct experimentation to fully understand the effect of a variant (Lee et al., 2014; XiangWei et al., 2019).

Experimental models, including cultured cells, *Drosophila*, zebrafish, and rodents such as mice and rats, are widely used to study epilepsy candidate genes. *Drosophila* is a particularly attractive model due to its short life cycle of only 50 days and its ability to produce a large number of offspring. Due to these factors, spatiotemporal genetic modifications, such as those enabled by the UAS/Gal4 system, can be used to conduct fly research in just a few months (Kepecs et al., 2002; Takai et al., 2020; Guerrini et al., 2021). The discovery of the *Drosophila* model is also easy to translate to other vertebrate models (Hunt et al., 2019; Hegazi et al., 2022).

Under various seizure-induction assays, adequate seizure-susceptible mutants of *Drosophila* adults, which correlate with different epilepsy subtypes, exhibit a wide spectrum of seizure-like behaviors. For instance, sudden unexpected death in epilepsy (SUDEP) mutants Shaker and Hyperkinetic display leg-shaking behavior following etherization (Kaplan and Trout, 1969; D’Adamo et al., 2020), while general epilepsy with febrile seizure plus (GEFG+) and Dravet syndrome (DS) knock-in mutations display loss of posture and random wing buzzing after heat application (Sun et al., 2012; Schutte et al., 2014), Na^+^ channel mutants display spontaneous shuddering (Kaas et al., 2016), whereas bang-sensitive (BS) mutants exhibit seizure-like activity when evoked mechanically. However, only one kind of activity, characterized by seizure and paralysis after a mechanical stimulus and observed in over twenty BS mutants, has been defined as “seizure-like” (Parker et al., 2011a). Therefore, we introduced two novel seizure phenotypes induced by vertex shock in *Drosophila* to extend the application of mechanical induction assays and further investigate the relationship between epileptic phenotypes and genotypes in both *Drosophila* and humans with greater precision.

However, a broad applicability screening system is urgently required since enormous epilepsy candidate genes must be verified by functional experiments. Here, we propose a set of simple experiments in *Drosophila* to evaluate seizure behaviors, brain morphology, and electrophysiology, which can expedite the progress of identifying epilepsy candidate genes. Especially, our approach introduces two novel seizure phenotypes induced by vertex shock and several optimized electrophysiological methods, thereby expanding the range of research methods available for investigating epilepsy in the *Drosophila* model.

## 2. Materials and methods

### 2.1. *Drosophila* stocks

The flies used in this study were fed standard cornmeal food and maintained in an incubator at 25°C (except for the special statement) and 60–70% humidity in a 12-h light-dark cycle. *UAS-Tango14-RNAi* (THU1886), *UAS-Sbf-RNAi* (THU0865), and *UAS-Cac-RNAi* (THU2586) flies were kindly donated by Tsing Hua Fly Center (https://thfc.zzbd.org/, Tsinghua University, Beijing, China). *UAS-Klp3A-RNAi* (BDSC: 43230) was purchased from Bloomington Fly Stock Center (Bloomington, IN, USA). *Canton-S* and *tub-Gal4* were kindly donated by Prof. LIU Ji-Yong (Guangzhou Medical University, Guangzhou, China). *UAS-EGFP*, *UAS-mCD8:GFP*, and *GH146-Gal4* were kindly donated by Prof. KE Ya (the Chinese University of Hong Kong, Hong Kong). *Canton-S* was used as the wild-type flies in this study.

### 2.2. Loss-of-function mutation model

The *tub-Gal4* line was utilized in the cross-breeding of two RNAi lines, the *Klp3A-RNAi* and the *Cac-RNAi*, to generate global knockdown of *Klp3A* and *Cac* in *Drosophila melanogaster* (*tub-Gal4* > *Klp3A-RNAi* and *tub-Gal4* > *Cac-RNAi*). The *elav-Gal4* line was utilized in the cross-breeding of two RNAi lines, the *Tango14*-RNAi and the *Sbf*-RNAi, to generate neuronal knockdown of *Tango14* and *Sbf* in *Drosophila melanogaster* (*elav-Gal4* > *Tango14-RNAi* and *elav-Gal4* > *Sbf-RNAi*). The efficiency of knockdown models was detected by qPCR.

### 2.3. Seizure behaviors

The seizure behavior test was performed on flies 3–5 days after eclosion. Flies were anesthetized with CO_2_ and transferred to another clean food vial 18–24 h before testing. Approximately two to seven flies were placed in one vial and mechanically stimulated with a vortex mixer (VWR, Radnor, PA, USA) at maximum speed for 20 s. To record the behavior of the flies, a high-resolution camera was utilized. Each genotype was evaluated using at least five trials, with each trial consisting of ten vials of flies. The rates of BS paralysis, which were recorded from the moment of “banging” until flies regained the ability to stand upright (Ganetzky and Wu, 1982), as well as those of late-phase seizure and hyperactive seizure in flies, were documented. The behavior assay was performed using a high-resolution camera (720–1080 P, 30–60 FPS at least), and the records were manually played back on the computer with video software (Baofeng Group Co., Ltd., China) to count the rates of BS.

### 2.4. Brain morphology

To examine morphological variations in the brain, *UAS-mCD8:GFP* was used to generate *tub-Gal4* > *UASmCD8:GFP; UAS-target gene-RNAi* knockdown flies and *tub-Gal4* > *UAS-mCD8:GFP* control flies, respectively, that were labeled with membrane GFP. The brain was dissected and fixed with 4% paraformaldehyde (PFA) in phosphate-buffered saline (PBS) with 0.1% Triton X-100 for 1 h at 25°C, then washed three times with 0.3% Triton X-100 PBS. Images were captured using a confocal microscope (SP8; Zeiss, Jena, Germany) and analyzed using the ImageJ software (National Institutes of Health, Bethesda, MD, USA).

### 2.5. Larvae development

The larval stage of flies can be divided into three molting stages, namely, the first, second, and third instar larvae. We utilized the length of fly larvae to evaluate their development. For the experiment, one female and three male flies were placed into a fresh food vial to cross for 24 h. After 24 h, the female flies were transferred to separate fresh food vials and allowed to lay eggs for 12 h (taking note of the exact time of the transfer), while the adult flies were removed. The vials containing the eggs were then incubated under standard conditions for varying durations in order to obtain larvae at the desired developmental stage. Once the larvae had reached the desired stage, 2 ml of a 20% sucrose solution was added to the food vials, and after 5 min, the floating larvae were decanted into a Petri dish. The larvae were then washed by transferring them to a dish containing 1X PBS using a pipette. To devitalize the larvae, they were heated in water bath equipment at 50°C for 10 min. After heating, the larvae were placed on a black card, and photographs of the larvae along with a scale were taken. The length of the larvae was then measured using the ImageJ NIH software.^1^

### 2.6. Electrophysiology

#### 2.6.1. Patch-clamp recording

Fly brains were dissected as previously described, transferred to a recording chamber with the fly external solution, and immobilized with a C-sharp holder (Gu and O’Dowd, 2006). The standard external solution contained (mM) 101 NaCl, 1 CaCl_2_, 4 MgCl_2_, 3 KCl, 5 glucose, 1.25 NaH_2_PO_4_, and 20.7 NaHCO_3_ (pH 7.2 and 250 mOsm). The internal solution for whole-cell recording contained (mM) 0.085 CaCl_2_, 1.7 MgCl_2_, 10 HEPES, 1 EGTA, 103 K-gluconate, 2 Na_2_-ATP, and 0.4 Na-GTP. The patch pipette for the attached recording was filled with an external solution. Recordings were acquired using the 700B amplifier, Digidata 1440B digital-analog converter, and pClamp 10.5 software (molecular devices).

#### 2.6.2. Evoked EPSP recording

The equipment, brain, and recording solution preparations used were identical to those for whole-cell recording. Evoked EPSPs between the antennal lobe (AL) and the mushroom body (MB) were stimulated by current pulses (0.5 ms; 0.1–0.5 mA) from the stimulation electrode on the antennal lobe (presynaptic structure). Then the evoked EPSPs were recorded by whole-cell recording on the MB neurons (postsynaptic structure) (Qiao et al., 2022b). Different intensities of current pulses were applied for establishing the input-output relationship. The subsequent recording was then performed using the current pulse to induce 50% of the maximum EPSP response. To test the synaptic connection, ten trials were performed with a 1-min interval between each trial. The latency and amplitude of evoked EPSPs were recorded and analyzed using the pClamp 10.5 software.

### 2.7. Statistical analysis

All quantitative data were presented as mean ± S.D. The Student’s *t*-test was used to compare two independent or paired samples. Multiple samples were analyzed by one-way ANOVA, and differences between the two groups were evaluated using Tukey’s *post hoc* test. Statistical analyses were performed with GraphPad Prism 7.00 and SPSS 20. The cutoff value for statistical significance was 0.05. The n number in the behavior data represented the number of trials.

## 3. Results

### 3.1. Seizure-like behavior under mechanical stimulation

#### 3.1.1. Hyperactivity seizure behavior

**FIGURE 1:**
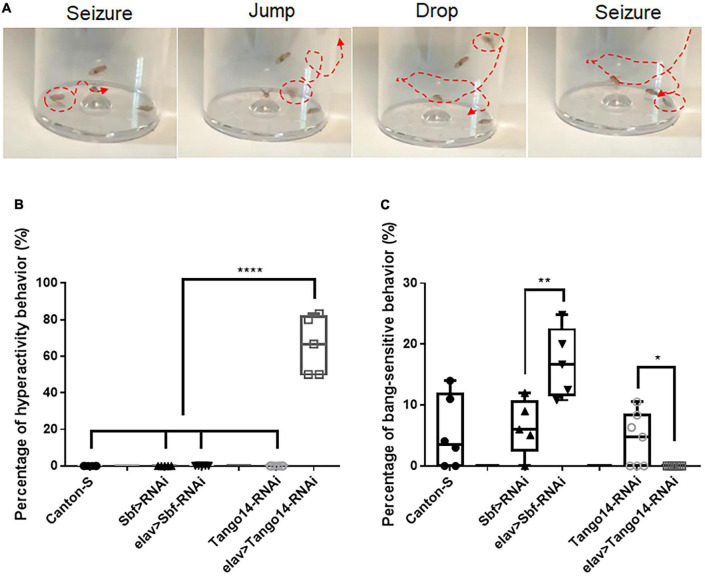
Hyperactivity seizure behavior in *Tango14* knockdown flies. **(A)** Typical hyperactivity seizure behavior is shown. The seizure stages contain the first-phase seizure stage, the jump-and-drop stage, and the second-phase seizure stage. **(B)** Approximately 70% of *Tango14* knockdown files (*elav* > *Tango14-RNAi*) showed hyperactivity seizure behavior. Contrarily, the control flies (*Canton-S* files and *Tango-RNAi* flies) did not show any hyperactivity seizure behavior. One-way ANOVA and Tukey’s multiple comparison tests. **(C)** *elav* > *Sbf-RNAi* showed a higher BS seizure rate than that of wild-type flies (*Sbf* > *RNAi*). While *elav* > *Tango14-RNAi* showed no classical BS seizure. **P < 0.01, ****P < 0.001.

Vortex stimulation is a simple and effective method to induce seizure behavior in *Drosophila*. Unlike classical BS seizure behavior (Parker et al., 2011a), our investigation of seizure sensitivity in the epilepsy candidate genes *Sbf* and *Tango14* revealed an unreported hyperactivity behavior. We administered the mechanical stimulation to flies with pan-neural knockdown of *Tango14* (*elav-Gal4* > *Tango14-RNAi*) and *Sbf* (*elav-Gal4* > *Sbf-RNAi*), as well as wild-type *Canton-S* flies. The *elav-Gal4* > *Sbf-RNAi* showed classical BS seizure behavior (^**^p = 0.0078, *n* > 5 in each group, one-way ANOVA, Tukey’s multiple comparisons tests, Figure 1C). While the *elav-Gal4* > *Tango14-RNAi* flies exhibited hyperactivity-like behavior that consisted of repeat drop and jump behaviors after seizure (Figure 1A and Supplementary video 1). Notably, this hyperactivity behavior, as defined in our study, differs from the previous definition, which is characterized by intense, uncoordinated motor activity before paralysis (Pavlidis and Tanouye, 1995). Flies typically recover quickly after a classical seizure-like behavior (Parker et al., 2011b; Wang et al., 2021), but in hyperactivity seizures, they repeatedly drop and jump, which may lead to recurring seizures. Such unique behavior was not observed in wild-type or mutant flies without mechanical stimulus. Approximately 66.00% of *elav-Gal4* > *Tango14-RNAi* flies showed this behavior, which cannot be observed in *elav-Gal4* > *Sbf-RNAi*, *Canton-S* WT files, and *Tango14-RNAi* flies (^****^p < 0.0001, *n* > 5 in each group, one-way ANOVA, Tukey’s multiple comparisons tests, Figure 1B).

#### 3.1.2. Late-phase seizure behavior

**FIGURE 2:**
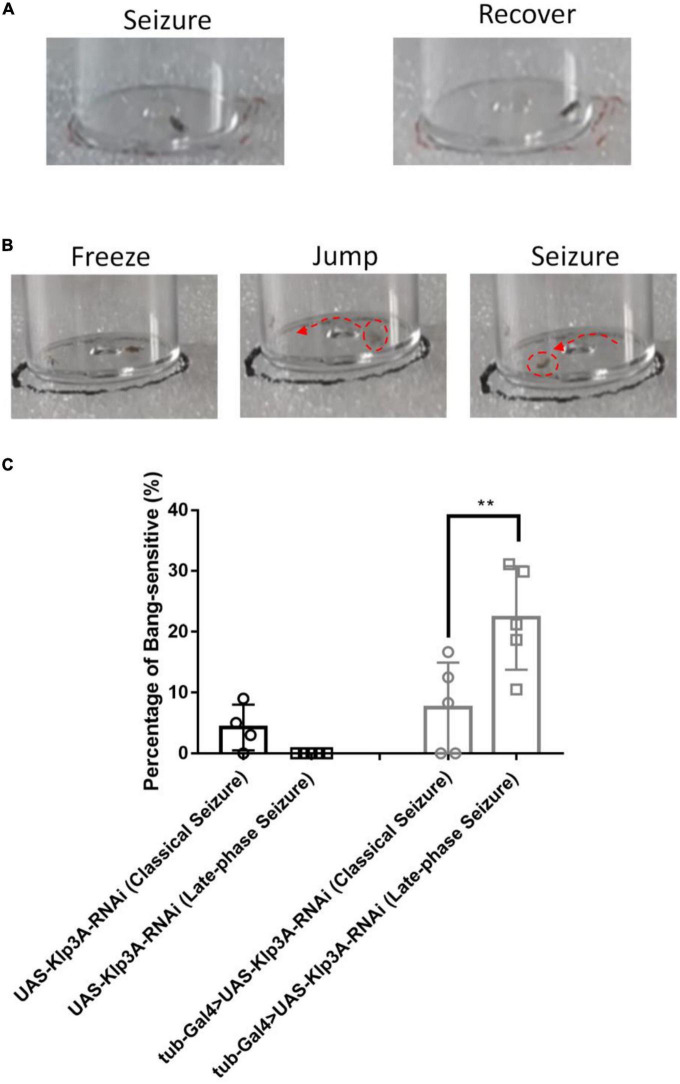
Late-phase seizure behavior is shown in Klp3A knockdown flies. **(A)** The flies showed seizure behavior immediately in classical seizure behavior. **(B)** Late-phase seizure behavior contained the freezing stage and jumping stage before seizure behavior. **(C)** *Klp3A* showed a higher rate in late-phase seizures than that in classical seizures. One-way ANOVA, Tukey’s multiple comparison tests. **P < 0.01.

In this study, another new type of seizure behavior called late-phase seizure was identified, which is distinct from the classical BS seizure behavior. Specifically, after vortex stimulation, the late-phase seizure behavior displayed a brief freezing or normal phase lasting 1–3 s before the onset of the seizure phase, as shown in Figure 2B and Supplementary video 2. In contrast, the classical BS seizure behavior started immediately after vortex stimulation without a freezing phase, as shown in Figure 2A. The knockdown of the epilepsy candidate gene *Klp3A* in flies (*tub-Gal4* > *UAS-Klp3A-RNAi*) resulted in a significantly higher occurrence of late-phase seizures (22.27 ± 8.49%, *n* = 5) compared to the *UAS-Klp3A-RNAi* flies (0.00 ± 0.00%, *n* = 5, *p* = 0.0002, one-way ANOVA, Tukey’s multiple comparisons test). However, there was no significant difference in the occurrence of classical seizures between the two groups (7.50 ± 7.45%, *n* = 5 vs. 5.67 ± 3.98%, *n* = 6, *p* = 0.95). Furthermore, the *tub-Gal4* > *UAS-Klp3A-RNAi* flies exhibited a significant difference in the manifestation of late-phase seizure and classical seizure (22.27 ± 8.49%, *n* = 5 vs. 7.50 ± 7.45%, *n* = 5, ^**^p = 0.0036, Figure 2C). These results suggest that the primary phenotype of *Klp3A* knockdown flies is late-phase seizure rather than classical or hyperactivity seizures.

### 3.2. Morphological observation in *Drosophila*

**FIGURE 3:**
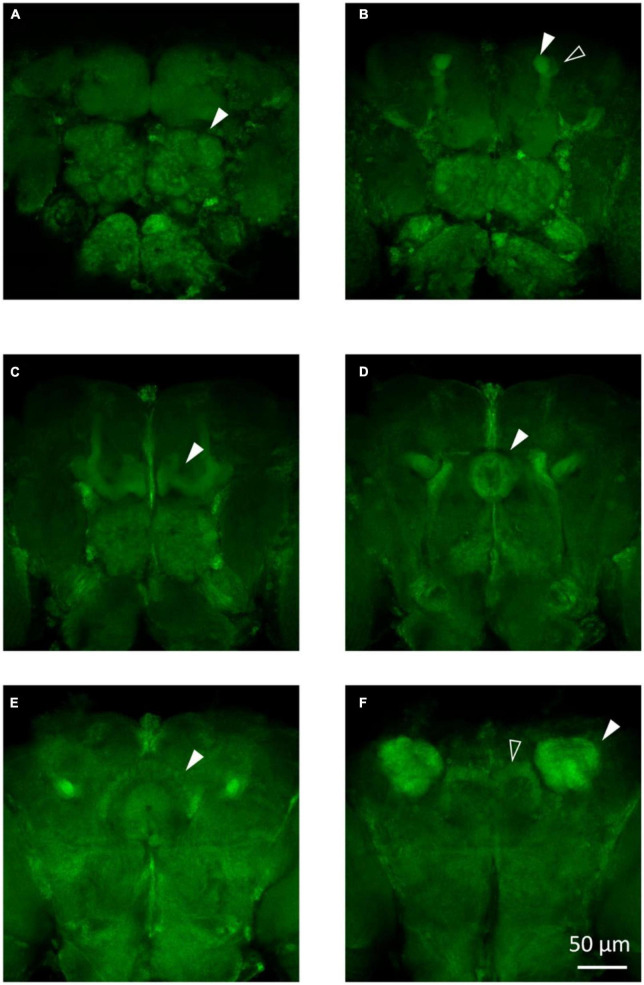
The main structures in the fly brain. **(A)** Antennal lobes are indicated by the arrow in the anterior view of the *tub-Gal4* > *UAS-mCD8:GFP* fly brain. **(B)** Alpha lobe (solid arrow) and alpha’ lobe (hollow arrow) of the mushroom body are shown in the anterior view of the fly brain. **(C)** Gamma lobe of the mushroom body in the *tub-Gal4* > *UAS-mCD8:GFP* fly brain. **(D)** Ellipsoid body of the central complex in the *tub-Gal4* > *UAS-mCD8:GFP* fly brain. **(E)** Fan-sharp body of the central complex in the *tub-Gal4* > *UAS-mCD8:GFP* fly brain. **(F)** Protocerebral bridge (hollow arrow) of the central complex and calyx (solid arrow) of the mushroom body are shown in the posterior view of the fly brain.

To visualize the major brain regions that can be affected by epilepsy, we utilized membrane GFP labeling *via* the *tub-Gal4* > *UAS-mCD8:GFP* construct. These regions, namely, the mushroom body and central complex (Figure 3), play a crucial role in higher-order cognitive functions. Our previous research has found that knockdown/knockout of genes involved in epilepsy or neural disorder, such as *UNC13B*, *LRP1*, and *YWHAZ*, leads to various brain abnormalities, including neuron blurring in mushroom body (Wang et al., 2021), partial destruction in the central complex (Zhang et al., 2022), and gamma lobe mutilation in the mushroom body (Wan et al., 2022). Although not all epilepsy gene defects could induce brain structural abnormalities, the morphological study still can provide valuable insights into the underlying mechanisms of epilepsy caused by specific genes.

### 3.3. Larvae development in *Drosophila*

**FIGURE 4:**
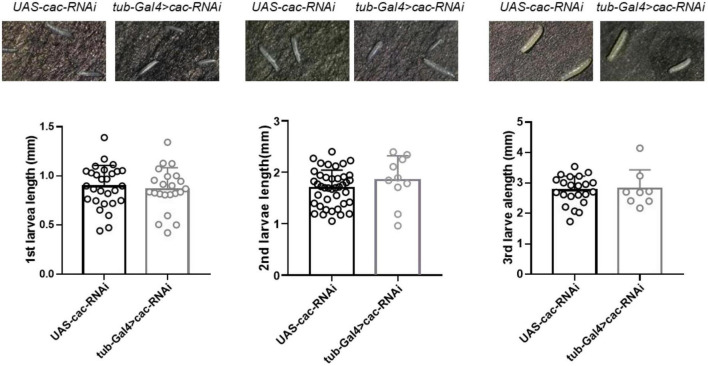
Global knockdown of calcium channel (cac) did not affect the development of *Drosophila*. The length of the 1st, 2nd, and 3rd larval stages did not show significant differences between wild-type files *UAS-Cac-RNAi* and calcium channel knockdown flies *tub-Gal4* > *cac-RNAi*.

*CACNA1A* is a well-known epilepsy-associated gene (OMIM*601011). To study the developmental effect of *CACNA1A*, we used the UAS/Gal4 system to establish calcium channel knockdown (KD) flies (tub-Gal4 > Cac-RNAi). UAS-cac-RNAi flies were used as wild-type (WT) flies The results showed that the global knockdown of *Cac* did not affect the development of 1st (WT: 0.89 ± 0.21, *n* = 28 vs. KD: 0.85 ± 0.22, *n* = 22, *p* = 0.64, Student’s *t*-test), 2nd (WT: 1.69 ± 0.35, *n* = 41 vs. KD: 1.85 ± 0.48, *n* = 10, *p* = 0.26), and 3rd (WT: 2.76 ± 0.47, *n* = 22 vs. KD: 2.81 ± 0.63, *n* = 8, *p* = 0.82) larval stage, compared to the wild-type flies (Figure 4).

### 3.4. EPSCs and action currents in the antennal lobe of *Drosophila*

**FIGURE 5:**
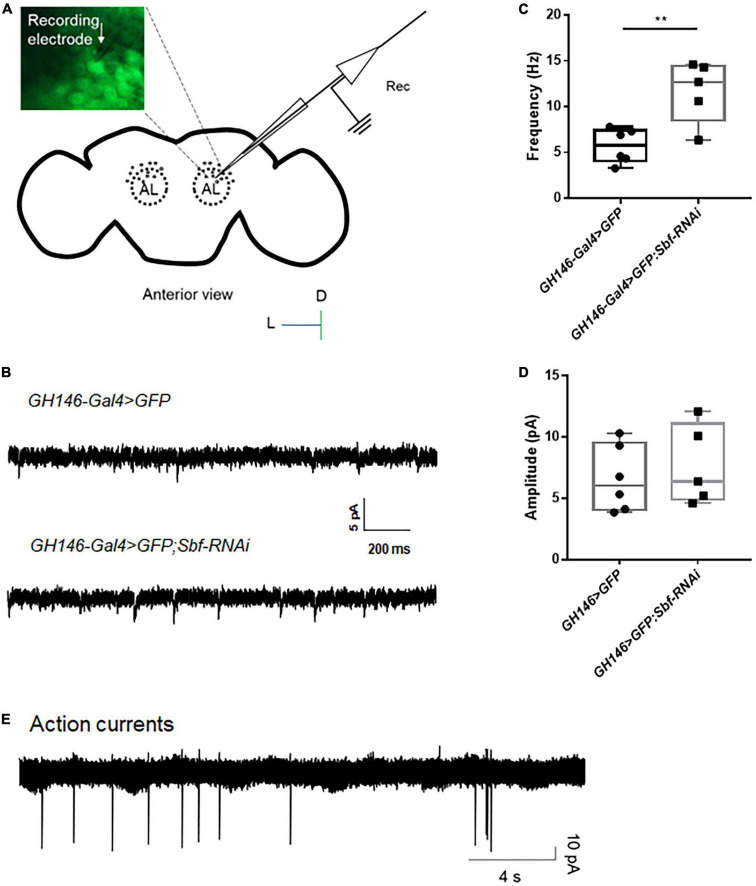
Whole-cell recording and attached recording in the fly brain. **(A)** Scheme of whole-cell recording or attached recording in the projection neurons of antennal lobes. Antennal lobes were observed in the anterior view of the brain. Neurons were labeled with GFP, and a recording electrode was used to patch the target neurons. **(B)** Typical trace of EPSCs in projection neurons of the wild-type flies and *Sbf* knockdown flies. The frequency of EPSCs in *GH146-Gal4* > *GFP;Sbf-RNAi* flies is higher than that in wild-type files (*GH146-Gal4* > *GFP*) **(C)**, but the amplitude of EPSCs showed no difference between knockdown flies and wild-type flies **(D)**. **(E)** Typical trace of action currents in the projection neurons. **P < 0.01.

The projection neuron located in the antennal lobe (AL) of the fly brain is a significant excitatory neuron group, and these neurons are easily distinguishable using bright field imaging in a patch microscope (Duan et al., 2012), which makes them an ideal model for investigating neuronal excitability in epilepsy-prone flies. Here, we performed whole-cell, voltage-clamp recordings on neurons in AL based on their expression of GFP (Figure 5A) to detect the excitatory postsynaptic currents (EPSCs) of *GH146-Gal4* > *GFP* WT flies. Typical traces of spontaneous EPSCs in WT flies *GH146-Gal4* > *GFP* are presented in Figure 5B. To test the setup in seizures, we established *Sbf*, another epilepsy candidate gene, knockdown flies *GH146-Gal4* > *GFP; Sbf-RNAi*. The *GH146-Gal4* > *GFP;Sbf-RNAi* flies had a significantly higher frequency of sEPSCs in projection neurons than that of *GH146-Gal4* > *GFP* WT flies (11.71 ± 3.39 Hz [*n* = 5] vs. 5.71 ± 1.86 Hz [*n* = 6]; ^**^p = 0.0046) (Figure 5C). There was no significant difference in sEPSC amplitude between *GH146-Gal4* > *GFP; Sbf-RNAi* and *GH146-Gal4* > *GFP* WT flies (6.33 ± 3.15 pA [*n* = 10] vs. 6.62 ± 2.70 pA [*n* = 6]; *p* = 0.85) (Figure 5D).

The attached recording is an essential electrophysiological technique for investigating neuronal excitability, particularly for analyzing spontaneous action potential currents (Dubin and Harris, 1997; Perkins, 2006). The recording captured and presented in Figure 5E displays the recorded action potential currents. Our previous study has shown that the knockdown of *UNC13B* induces a higher frequency of action currents in projection neurons (Wang et al., 2021).

### 3.5. Evoked EPSP from the antennal lobe to the mushroom body of *Drosophila*

**FIGURE 6:**
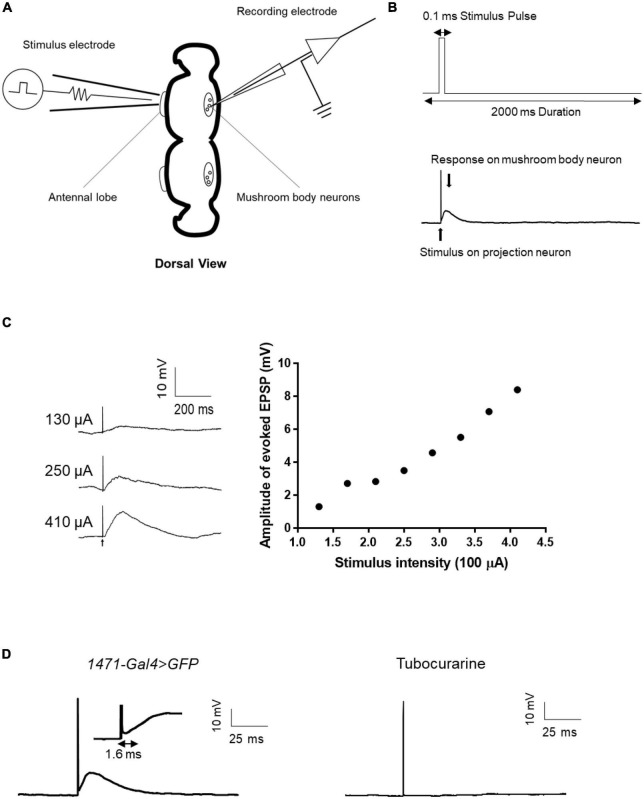
Evoked EPSP recording in the fly brain. **(A)** Scheme of evoked EPSP recording between the antennal lobe and mushroom body. The stimulation electrode attached to the antennal lobe and recording electrode was used to record evoked EPSP in mushroom body neurons. **(B)** Stimulus protocol for the stimulation electrode is shown in the upward picture. Stimulus artifact and response signal are shown in the downward picture. **(C)** Typical trace of evoked EPSP in different stimulus intensities and the input-output curve of evoked EPSP. **(D)** Latency time of evoked EPSP in gamma neuron. Tubocurarine confirmed that the evoked EPSP is cholinergic.

Given the numerous genes related to synaptic structure that has been implicated in epilepsy (Lammertse et al., 2020), it is evident that synaptic connections must be considered when evaluating the mechanisms of epilepsy candidate genes. However, to date, few epilepsy gene studies conducted using the *Drosophila* model have utilized electrophysiological techniques to directly assess synaptic events. To address this knowledge gap, we established a single-cell-level recording technique to monitor evoked excitatory postsynaptic potentials (EPSPs) from the antennal lobe (AL) to the mushroom body (MB), which is a major synaptic functional connection in the *Drosophila* central nervous system. The technique involved forming a whole-cell recording on an MB neuron and applying an electrical stimulus to AL neurons to induce evoked EPSPs from AL to MB (Figures 6A, B). The amplitude of the evoked EPSPs increased with increasing stimulus intensity (Figure 6C). The input-output relationship was established to determine the appropriate stimulus intensity, and the current intensity that induced 50% of the maximum EPSP response was used for subsequent recordings. Tubocurarine, a cholinergic receptor inhibitor, was used to confirm the functional connection between AL and MB (Parker et al., 2011b). The latency of the evoked EPSPs varied depending on the different connections between projection neurons and mushroom body neurons. For example, gamma neurons (*1471-Gal4* > *GFP*) exhibited evoked EPSPs with a latency of approximately 1.6 ms (Figure 6D).

### 3.6. Knockdown efficiency detected by RT-qPCR

**FIGURE 7:**
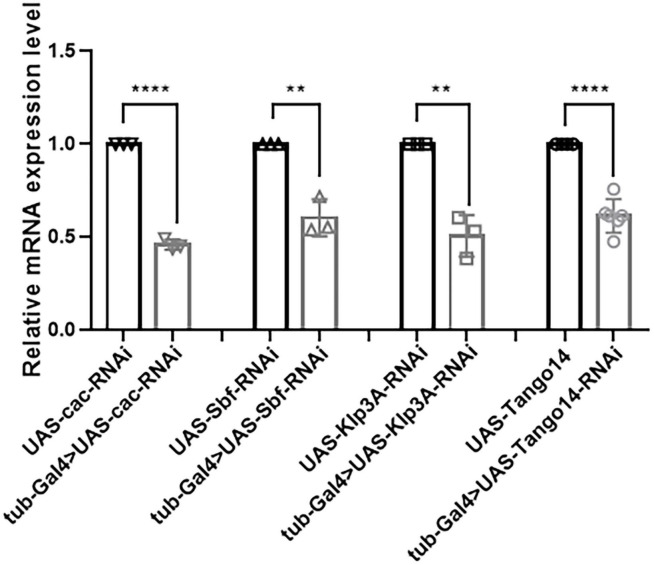
Knockdown efficacy of knockdown fly lines. Relative expression of mRNA in *Cac* knockdown flies, *Sbf* knockdown flies, *Klp3A* knockdown flies, *Tango14* knockdown flies, and wild-type flies. **P < 0.01, ****P < 0.001.

In terms of knockdown efficiency, *tub-Gal4* > *Cac-RNAi, tub-Gal4* > *Sbf-RNAi, tub-Gal4* > *Klp3A-RNAi*, and *tub-Gal4* > *Tango14-RNAi* had values of 53.95, 39.53, 49.26, and 38.59%, separately [46.05 ± 2.76% (*n* = 3) vs. 100 ± 0.00% (*n* = 3), ^****^p < 0.0001; 60.47 ± 9.86% (*n* = 3) vs. 100 ± 0.00% (*n* = 3), ^**^p = 0.0023; 50.74 ± 11.19% (*n* = 3) vs. 100 ± 0.00% (*n* = 3), ^**^p = 0.0016; 61.41 ± 8.99% (*n* = 6) vs. 100 ± 0.00% (*n* = 6), ^****^p < 0.0001, Figure 7]. *Cac, Sbf, Klp3A, and Tango14* were knockdown successfully in these *Drosophila* lines, as shown in Figure 7.

## 4. Discussion

Trio-based WES has been successful in defining the most likely epilepsy-implicated loci and screening out candidate genes, such as *UNC13B, CELSR3*, and *CDK19* in numerous studies (Chung et al., 2020; Wang et al., 2021; Li et al., 2022). Besides, NGS analysis of a large cohort of patients with epilepsy revealed that approximately 14% of sequence changes in 70 common and rare mutations were detected as nonsense or deletion (Lindy et al., 2018), while missense or frameshift mutations can also lead to loss-of-function (LOF) consequences (Chen et al., 2017; Lindy et al., 2018). The Gal4/UAS system allows for precise spatiotemporal manipulations of gene expression for cell labeling or gene function analysis (Del Valle Rodríguez et al., 2011). By crossing Gal4 driver lines to UAS-RNAi lines, the expression of UAS-linked genes can be effectively blocked in the chosen Gal4 pattern. However, some missense mutations lead to gain-of-function (GOF). For example, hundreds of missense mutations, including GOF mutations, in the gene *SCN1A* can confer a wide spectrum of epileptic phenotypes in *Drosophila* as they do in humans (Schutte et al., 2014), thus knock-in *Drosophila* lines mimicking the missense variants should be generated to realize more precise genetic editing (Xue et al., 2014). In our previous study, c.147A > T missense variation of *Ywhaz* was created using the CRISPR/Cas9 system to confirm its pathogenic effects (Wan et al., 2022). Thus, the knockdown model established by the Gal4/UAS system and the knock-in model established by the CRISPR/Cas9 system can cover most scenarios of gene mutations, including LOF and GOF mutations.

Animal experimentation can be a challenging and time-consuming process for validating the extensive range of candidate genes and variants proposed by WES. However, *Drosophila* has emerged as an attractive model organism to examine human epilepsy due to its short life span, strong genomic conservation, the abundance of technological resources for genomic manipulations (Shulman, 2015), and the exhibition of seizure behavior similar to humans. Historically, mechanical induction assays in *Drosophila* have only elicited one seizure behavior. This study expands the range of seizure-like phenotypes in the *Drosophila* model by discovering two novel seizure behaviors. Knockdown of *Tango14* results in hyperactive seizure behavior (Figure 1), while knockdown of *Klp3A* results in late-phase seizure behavior (Figure 2). Our findings were further validated by the demonstration of hyperactivity seizure behavior in knockdown of the *Ywhaz* gene (Wan et al., 2022), suggesting that this novel behavior could be applied to verifying other epilepsy candidate genes. These results imply that loss of function in different candidate genes can result in distinct phenotypes. In light of our observation, the hyperactivity seizure, manifesting increased movement and excitement, is comparable to hyperesthesia and highly consistent with the hyperactive-automatism subtype in complex partial epilepsy. The late-phase seizure may correlate with focal epilepsy originating from the sensory cortex. However, further investigation into the genotype-phenotype relationship in *Drosophila* is required through additional studies.

Electrophysiology has shown to be a valuable tool for the study of epilepsy, with numerous studies reporting its effectiveness in assessing electrical activity in the giant fiber and neuromuscular junction (NMJ) (Kuebler et al., 2001; Marley and Baines, 2011; Parker et al., 2011a,b; Duan et al., 2012; Xue et al., 2014). However, its application in the central nervous system (CNS) studies of epilepsy using the *Drosophila* model is less common (Guo et al., 2019; Savitsky et al., 2020; Chi et al., 2022). Our study adopted three electrophysiological methods, namely, whole-cell recording, cell-attached recording, and evoked EPSP recording, to directly explore the electrophysiological characteristics in the CNS. Whole-cell/cell-attached recordings allow us to assess the neuronal excitability of mutants compared to wild-type neurons in specific GFP-expressed regions of the *Drosophila* brain, such as the mushroom body (MB), a major high-level brain structure in flies. We could determine whether the cells are firing action currents and record the firing modes, such as tonic or burst firing. Moreover, the approach of recording evoked EPSP from AL to MB, previously used to reveal input-timing-dependent plasticity in the MB circuit during olfactory learning (Qiao et al., 2022b), has the potential to investigate the synaptic and molecular causes of epilepsy. The latency of evoked EPSP can measure the time interval of monosynaptic transmission in target synapses (Gil and Amitai, 1996). Our preliminary results suggest that the latency of evoked EPSP in PN-gamma neurons is around 1.6 ms (Figure 6D), validating the efficacy of our techniques in accurately detecting the time interval of target synaptic transmission. While we have yet to observe positive results in any epilepsy candidate genes, these methods can provide a strong tool for studying genes involved in synaptic function coding in epilepsy. Combining these electrophysiological methods with the LPF recording technique in the CNS of *Drosophila* (Iyengar and Wu, 2021) can develop an instrumental platform for investigating the cellular and synaptic mechanisms underlying neural function or dysfunction (Roemmich et al., 2018), with excellent accessibility for studying epilepsy’s pathological and pharmacological aspects (Sheeba et al., 2008).

The prevalence of seizures and epilepsies is alarmingly high among infants and preschoolers, with 10–25% of these young patients suffering from intractable seizures and varying degrees of intellectual/developmental disabilities (ID/DD) (Chung et al., 2020). Understanding the genetic basis of these conditions is crucial for accurate diagnosis, prognosis, genetic counseling, and treatment. Recent advances in NGS have revealed that *de novo* missense mutations in the *CACNA1A* gene contribute to ID/DD and therapy-resistant epilepsy (Indelicato and Boesch, 2021). As a result, our development study utilized the knockdown of the *Cac* gene (the ortholog of *CACNA1A*) to further investigate the impact of epilepsy candidate genes on development. Although approximately 50% knockdown did not demonstrate a significant effect (Figure 7), the study provided a reliable protocol for examining the impact of epilepsy candidate genes on development. The use of Gal4 drivers in conjunction with Gal80ts, which exhibit minimal activity at 18^°^C and maximal activity at 29^°^C (Duffy, 2002), can precisely restrict the gene knockdown to the first, second, and third instar larval or pupal stages through temperature control. By measuring parameters such as larval or pupal weight, pupation rate, eclosion rate, and body length (Zhang et al., 2021), we can conduct a more comprehensive analysis of candidate genes that exclusively express during specific developmental stages.

The potential of the genetic screening system, which models epilepsy using genotypic and phenotypic strategies, could be further demonstrated by applying it to a larger collection of genes implicated in epilepsy, such as a gene set. In addition, it is important to consider the sex of the flies in current experiments, as gender differences in epilepsy exist in both humans and *Drosophila* (Sharma et al., 2009).

Notably, epilepsy in individuals may be caused by multiple pathogenic variants, both common and rare, with a range of effect sizes (Ferraro and Buono, 2006; Epi25 Collaborative, 2019; Thijs et al., 2019). *Drosophila* is advantageous in the study of epilepsy due to its ability to map genetic interactions, which is facilitated by powerful resources and techniques such as genome-wide RNAi transgenic stocks (Dietzl et al., 2007; Ni et al., 2011), collections of mapped transposon insertion alleles, Gal4/UAS system, and the recently developed CRISPR/Cas9 system. Notably, integrating the RNAi with Gal4/UAS system could achieve double- or even triple-knockdown in one generation without complicated genetic crosses (Okumura et al., 2015). *Drosophila* is a premier genetic model system for elucidating mechanisms responsible for many neurological genetic disorders (Jackson et al., 1998; Finelli et al., 2004). By making the most of resources and strategies, *Drosophila* holds great potential for advancing our understanding of the pathogenesis of epilepsy and other complex polygenic disorders, as well as the development of potential therapies.

## Data availability statement

The original contributions presented in this study are included in the article/Supplementary material, further inquiries can be directed to the corresponding author/s.

## Author contributions

C-QL, J-DQ, and K-CW designed the study and wrote the manuscript. X-CQ, M-FH, D-HL, C-QL, S-MX, X-XZ, Y-ML, and W-JZ performed the behavior experiments. J-DQ performed the electrophysiological experiments. All authors contributed to the article and approved the submitted version.

## Footnotes

1
https://imagej.nih.gov/ij/
